# Etiologies and clinical characteristics of young patients with angle-closure glaucoma: a 15-year single-center retrospective study

**DOI:** 10.1007/s00417-021-05172-6

**Published:** 2021-04-19

**Authors:** Feng Gao, Jiajian Wang, Junyi Chen, Xiaolei Wang, Yuhong Chen, Xinghuai Sun

**Affiliations:** 1 Department of Ophthalmology & Visual Science, Eye & ENT Hospital, Shanghai Medical College, Fudan University, 83 Fenyang Road, Shanghai, 200031 China; 2 NHC Key Laboratory of Myopia, Chinese Academy of Medical Sciences, and Shanghai Key Laboratory of Visual Impairment and Restoration (Fudan University), Shanghai, China; 3 State Key Laboratory of Medical Neurobiology and MOE Frontiers Center for Brain Science, Institutes of Brain Science, Fudan University, Shanghai, China

**Keywords:** Angle-closure glaucoma, Etiologies, Young patients, Chinese

## Abstract

**Purpose:**

To investigate the etiologies and the clinical characteristics of angle-closure glaucoma (ACG) patients younger than 40 years old in Chinese.

**Methods:**

Inpatients with diagnosis of ACG and diagnosed age younger than or equal to 40 years old, who were admitted in Eye, Ear, Nose, and Throat Hospital Fudan University from 2002 to 2017, were included in this retrospective non-comparative case series. The underlying causes and clinical features for all the patients were analyzed by comprehensive review of medical charts.

**Results:**

A total of 298 patients (463 eyes) met the criteria, including 153 females (51.3%) and 145 males (48.7%); the mean age was 25.6 ± 13.0 years. Primary angle-closure glaucoma (PACG), uveitis, and anterior segment dysgenesis (ASD) were the top three etiologies in our patients, which accounted for 32.6%, 20.3%, and 15.1% of the total patients respectively. PACG mainly occurs after 30 years of age and ASD is the top reason of ACG in patients younger than 20 years old. Other known etiologies include iridocorneal endothelial syndrome, neovascular glaucoma, nanophthalmos, retinitis pigmentosa, spherophakia, bestrophinopathy, persistent fetal vasculature, iridociliary cysts, congenital retinoschisis, Marfan’s syndrome, retinopathy of prematurity, familial exudative vitreoretinopathy, congenital retinal folds, Coat’s disease, and neurofibromatosis.

**Conclusions:**

We described the uncommon presentation of ACG in Chinese young patients. Although unusual, most of the etiologies could be identified. Therefore, more careful and comprehensive examinations are needed for early detection and timely treatment for young ACG patients.
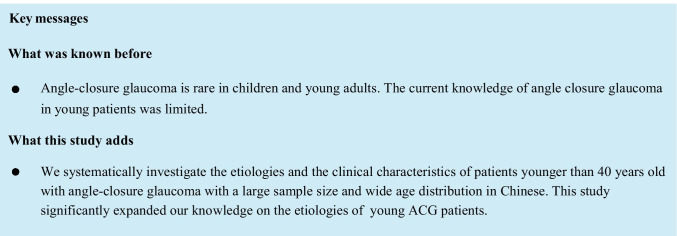

**Supplementary Information:**

The online version contains supplementary material available at 10.1007/s00417-021-05172-6.

## Introduction

Glaucoma, characterized by retinal ganglion cell degeneration with or without intraocular pressure elevation, is the leading cause of global irreversible blindness. It is estimated that the number of glaucoma patients aged over 40 years old was 60.5 million in 2010, and was predicted to increase to 79.6 million in 2020 and 111.8 million in 2040 [1, 2]. Although primary open-angle glaucoma (POAG) is the most common type, the distribution of glaucoma types varies among races and areas. Asian accounts for 87% of worldwide primary angle-closure glaucoma (PACG) cases [1] and China has the biggest population of PACG in the world. In a population survey by Foster and Johnson [3], 1.7 million glaucoma adults suffered from bilateral blindness in China, among whom 91% were caused by PACG. Thus, PACG poses a major public health problem with significant economic and social impact in Asia especially in China.

Advancing age is acknowledged as a significant risk factor for PACG, almost all of the epidemiological studies on PACG were carried out in adults over age of 40 years old [1–3].

That is because angle-closure glaucoma (ACG) is rare in children and young adults. According to an investigation about age-specific prevalence, the peak age of PACG was around 60 to 80 years old and the prevalence among those older than 60 years of age was about tenfold higher than people aged younger than 40 years old [4].

Thus, young patients presented as ACG are likely to have different etiologies behind. The underlying causes that make them develop ACG are not very well understood. Although some of them are still PACG, various etiologies could mimic the presentation of PACG and need to be differentiated. To our knowledge, except for a few case reports [5–7], there was only one study reviewing the etiologies of young patients with angle closure [8], which provided an incomplete picture of young ACG patients due to small sample size. Since young ACG patients have different clinical manifestations and prognosis, comprehensive and in-depth understanding of the etiologies could help to make accurate diagnoses and provide better treatment. Thus, we meant to systematically investigate the etiologies and the clinical characteristics of patients younger than 40 years old with ACG with a larger sample size and wider age distribution in Chinese by comprehensive review of medical charts.

## Methods

This retrospective study was approved by the ethics committee of Eye, Ear, Nose, and Throat Hospital, Fudan University. The study protocol adhered to the tenets of the Declaration of Helsinki.

### Study population

Patients with diagnosis of ACG and with diagnosed age younger than or equal to 40 years old, who were admitted in Eye, Ear, Nose, and Throat Hospital, Fudan University, from October 2002 to September 2017, were included. ACG was defined as having glaucomatous optic neuropathy due to high intraocular pressure (IOP) caused by angle closure based on gonioscopy or ultrasound biomicroscopy (UBM). PACG was defined as primary angle closure (PAC) together with evidence of glaucomatous optic neuropathy [9]. For subjects diagnosed with secondary ACG, only eyes with 180° or more peripheral anterior synechiae (PAS) were included. Patients with histories of ocular trauma and surgeries (except glaucoma surgeries) were excluded.

### Data collection

We extracted the following information from the inpatient medical records: (1) general information: name, gender, date of birth, diagnosed age, diagnosis, underlying etiology or clinical status, and family histories; (2) biological data: visual acuity, IOP (initial and final), corneal diameter, anterior chamber depth (ACD), lens thickness (LT), anterior chamber angle status and iris morphology (by gonioscopy or UBM), axial length (AL), the fundus assessment, and optic disc evaluation; (3) therapeutic intervention: treatment of medicine, laser or surgery, and the prognosis of patients. After reviewing all the information, we concluded the diagnosis, the underlying causes, and clinical features for each patient.

## Results

**Fig. 1:**
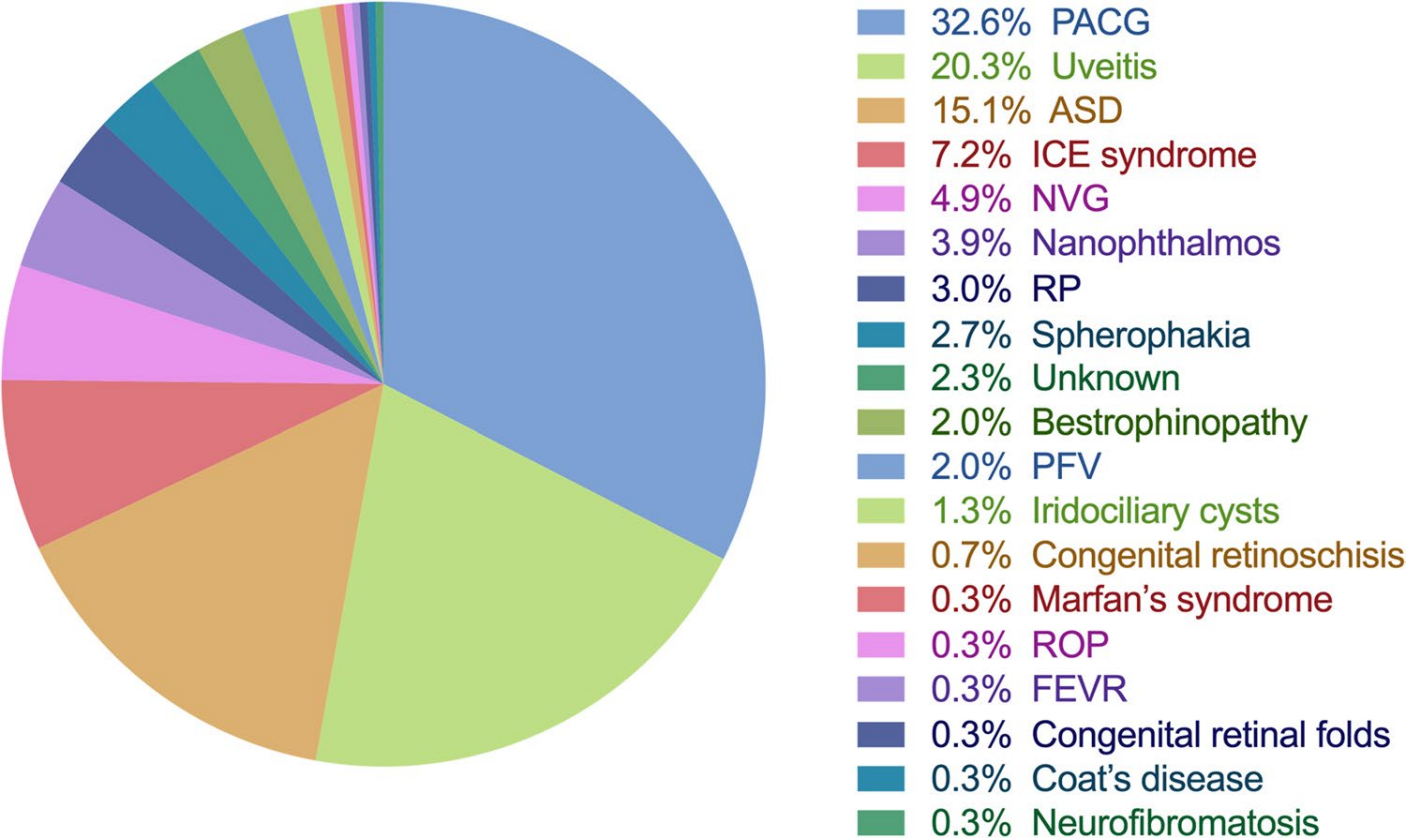
The etiologies of angle-closure glaucoma and their respective proportions in our patients. PACG, primary angle-closure glaucoma; ASD, anterior segment dysgenesis; ICE, iridocorneal endothelial; NVG, neovascular glaucoma; RP, retinitis pigmentosa; PFV, persistent fetal vasculature; ROP, retinopathy of prematurity; FEVR, familial exudative vitreoretinopathy. The proportions were analyzed based on individuals. Six patients were enrolled in two different groups: they all had PACG in one eye, but uveitis (1 patient), ICE syndrome (1 patient), NVG (1 patient), and nanophthalmos (3 patients) in the fellow eye. They were counted 0.5 in each group respectively for statistical analysis

In total, 298 patients (a total of 463 eyes), including 153 females (51.3%) and 145 males (48.7%), met the inclusion criteria, as shown in Fig. 1 and Table 1. The mean age at diagnosis of ACG in our patients was 25.6 ± 13.0 years (range, 0.1–40 years). The distribution of age at diagnosis was that 52 patients (17.4%) aged from 0 to 10 years old, 41 patients (13.8%) aged from 11 to 20 years old, 65 patients (21.8%) aged from 21 to 30 years old, and 140 patients (47.0%) aged from 31 to 40 years old respectively. One hundred fifty-eight patients (53.0%) were under 30 years old.

**Table 1:** The demographic information of our patients

Diagnosis	Number of patients	Age, mean ± SD (range), years	Gender female/male	Affected eyes unilateral/bilateral
PACG	100	34.4 ± 5.1 (13–40)	68/32	16/166
Uveitis	61	23.8 ± 12.8 (0.25–40)	20/41	47/28
ASD	45	11.5 ± 12.6 (0.13–38)	14/31	21/48
ICE syndrome	22	33.2 ± 6.3 (14–40)	14/8	20/4
NVG	15	20.1 ± 12.2 (0.1–40)	5/10	15/0
Nanophthalmos	13	29.5 ± 8.8 (13–40)	6/7	6/14
RP	9	29.7 ± 7.0 (18–39)	4/5	0/18
Spherophakia	8	13.4 ± 5.6 (6–20)	4/4	1/14
Bestrophinopathy	6	26.2 ± 6.4 (18–34)	5/1	0/12
PFV	6	19.9 ± 16.4 (0.3–39)	3/3	4/4
Iridociliary cysts	4	24 ± 14.0 (8–40)	3/1	3/2
Congenital retinoschisis	2	19 ± 1.4 (18–20)	0/2	0/4
Marfan’s syndrome	1	40	1/0	0/2
ROP	1	17	1/0	1/0
FEVR	1	12	0/1	1/0
Congenital retinal folds	1	30	1/0	1/0
Coat’s disease	1	3	0/1	1/0
Neurofibromatosis	1	4	0/1	1/0

*PACG*, primary angle-closure glaucoma; *ASD*, anterior segment dysgenesis; *ICE*, iridocorneal endothelial; *NVG*, neovascular glaucoma; *RP*, retinitis pigmentosa; *PFV*, persistent fetal vasculature; *ROP*, retinopathy of prematurity; *FEVR*, familial exudative vitreoretinopathy. Seven patients (6 females and 1 male) were unknown due to incomplete information

The top one cause of ACG in young patients was still PACG. A total of 182 eyes of 100 patients (68 females and 32 males) accounted for 32.6% of total patients and 39.3% of total eyes in our series. The mean age at diagnosis of PACG in our patients was 34.4 ± 5.1 years (range, 13–40 years). The distribution of age was 2 patients (2.0%) aged from 11 to 20 years old, 17 patients (17.0%) aged from 21 to 30 years old, and 81 patients (81.0%) aged from 31 to 40 years old respectively. With respect to the mechanisms of angle closure, 50 PACG patients (50.0%) were caused by multiple mechanisms, 25 patients (25.0%) were diagnosed as plateau iris syndrome (PIS), 11 patients (11.0%) were caused by pure pupillary block, and 14 patients (14.0%) were unknown due to incomplete information. Eighty subjects were recorded family history information. Twenty-four patients (30.0%) had glaucoma family histories, among whom 17 (70.8%) were parents. The mean AL was 21.66 ± 1.03 mm (range, 20.00–26.09 mm). The mean ACD was 1.89 ± 0.30 mm (range, 1.27–2.96 mm) and the mean LT was 4.36 ± 0.36 mm (range, 3.63–5.44 mm).

Among the 182 PACG eyes, 157 eyes had operation records. One hundred fourteen eyes (72.6%) of 63 patients (63.0%) underwent only one operation: trabeculectomy with or without mitomycin C (69.0%), laser peripheral iridotomy or surgical peripheral iridectomy ( 25.7%), or phacoemulsification (phaco) and intraocular lens (IOL) implantation with or without goniosynechialysis (4.4%). Twenty-eight eyes (17.8%) of 25 patients (25.0%) underwent two operations, and 15 eyes (9.6%) of 12 patients (12.0%) were subjected to multiple surgeries, including trabeculectomy, phaco and IOL implantation, ExPress or Ahmed tube implantation, or pars plana vitrectomy (PPV). Malignant glaucoma (MG) was the most significant postoperative complication in our patients and all occurred after trabeculectomy. Nineteen eyes (12.1%) of 16 patients (16.0%) suffered from MG, including 10 females and 6 males, the mean age was 33.8 ± 6.2 years (range, 22–40 years), and the mean AL was 21.29 ± 0.82 mm (range, 20.23–22.75 mm). The initial PPV was successful in 17 eyes (89.5%), and additional vitrectomy surgeries were performed in the remaining two eyes.

The second leading cause was uveitis. In our study, 75 eyes (16.2%) of 61 patients (20.3%) with uveitis developed ACG, and about two-thirds of whom were male. The mean age at diagnosis of this group of patients was 23.8 ± 12.8 years (range, 0.25–40 years). The mean AL was 24.04 ± 1.95 mm (range, 19.00–27.95 mm) and the mean ACD was 2.57 ± 0.64 mm (range, 0.73–3.87 mm). Because of incomplete information, the specific clinical types of uveitis were unclear except 2 females (4 eyes) diagnosed with Vogt-Koyanagi-Harada (VKH) syndrome and 1 male (1 eye) diagnosed with ocular toxocariasis.

Anterior segment dysgenesis (ASD) was the third common diagnosis in our study. Sixty-nine eyes (14.9%) of 45 ASD patients (15.1%) were included. They all had extensively closed anterior chamber angle and elevated IOP. The mean age at diagnosis of ACG patients due to ASD was 11.5 ± 12.6 years (range, 0.13–38 years) and 73.4% of the patients aged younger than 20 years old. The mean AL was 24.47 ± 2.87 mm (range, 20.29–31.77 mm) and the mean ACD was 2.49 ± 0.78 mm (range, 0.66–4.64 mm). Except for 17 unclassified ASD, Axenfeld-Rieger (A-R) syndrome (16 patients) was the most common type in this group of patients, followed by Peter’s anomaly (4 patients), microcornea (4 patients), aniridia (2 patients), and congenital ectropion uvea (2 patients). Of the 17 unclassified ASD, 14 were within 1-year-old and their clinical information was limited.

In addition, ACG was also found in the following diseases with each accounting for approximately 1 to 7% of total (Fig. 1 and Table 1). Twenty-two patients (24 eyes) had iridocorneal endothelial (ICE) syndrome. Fifteen patients (15 eyes) had unilateral neovascular glaucoma (NVG). Among them, four eyes were retinal vein occlusion (RVO), three eyes were proliferative diabetic retinopathy (PDR), one eye was retinal detachment (RD), and one eye was Sturge-Weber syndrome, and the remaining six eyes were unknown. Thirteen patients (20 eyes) were diagnosed as nanophthalmos by the criteria of AL ≤ 20.0 mm without morphologic malformation [10]. The mean AL was 18.03 ± 1.63 mm (range,14.57–19.92 mm) and the mean ACD was 1.77 ± 0.73 mm (range, 0.12–3.03 mm) for this group of patients. Nine patients (18 eyes) had bilateral retinitis pigmentosa (RP). Eight patients (15 eyes) had spherophakia with the mean lens thickness of 4.43 ± 0.27 mm (range, 4.27–4.97 mm). Six patients (12 eyes) had bestrophinopathy, including 4 patients diagnosed as autosomal recessive bestrophinopathy (ARB) with homozygous or compound heterozygous mutations in *BEST1* gene and 2 patients diagnosed as Best disease with heterozygous mutations in *BEST1* gene. Six patients (eight eyes) had persistent fetal vasculature (PFV). Four patients (five eyes) had multiple iridociliary cysts. And seven patients (nine eyes) were unknown due to incomplete information.

The rest of the patients harbored diagnoses accounting for less than 1% of total respectively. They were congenital retinoschisis (two patients, four eyes), Marfan’s syndrome (one patient, two eyes), retinopathy of prematurity (ROP) (one patient, one eye), familial exudative vitreoretinopathy (FEVR) (one patient, one eye), congenital retinal folds (one patient, one eye), Coat’s disease (one patient, one eye), and neurofibromatosis (one patient, one eye).

## Discussion

Age is known to be strongly associated with PACG. Majority of PACG patients are older than 40 years old. With aging, the increase of lens thickness and the decrease of the anterior chamber depth lead to the anterior chamber angle becoming narrower or even closed, which significantly increase the incidence of PACG. However, cases younger than 40 years old who present ACG are also seen occasionally. A number of other diseases could have similar manifestations to PACG but with completely different responses and prognosis to treatment. There are limited studies about the etiologies of angle closure in young patients, most of them were scattered cases except Ritch et al. evaluated some causes in patients within 40 years old [8]. In this study, we reviewed 15 years’ inpatients records and included 298 ACG patients younger than 40 years old, which has a stricter definition of angle closure, much larger sample size, and more comprehensive etiologies than the study Ritch et al. carried out [8] (Table 2). In total, 18 etiologies were discovered in our study compared to 10 etiologies in Ritch’s study. Eight identical causes are as follows: PIS, uveitis, iridociliary cysts, ROP, PHPV, nanophthalmos, Marfan syndrome, and Weill-Marchesani syndrome. In addition, we found ASD, ICE syndrome, NVG, RP, bestrophinopathy, congenital retinoschisis, FEVR, congenital retinal folds, Coat’s disease, and neurofibromatosis were possible causes of young ACG as well.

**Table 2:** The comparison of basic information of angle closure in young patients

	Number of patients	Female/male No. (%)	Age at diagnosis, mean ± SD (range), years	Number of etiologies	The most common diagnosis (no. %)
Our patients	298	153 (51%)/145 (49%)	25.6 ± 13.0 (0.1–40)	18	PACG (100, 32.6%)
Ritch et al	67	49 (73%)/18 (27%)	31.3 ± 8.5 (3–40)	10	PACG (37, 55.2%)

*PACG*, primary angle-closure glaucoma. Six patients were enrolled in two different groups: they all had PACG in one eye, and uveitis (1 patient), ICE syndrome (1 patient), NVG (1 patients), and nanophthalmos (3 patients) in the fellow eye. They were counted 0.5 in each group respectively for statistical analysis

In our retrospective study, we found PACG (32.6%), as the leading diagnosis, was female-dominated and the mean age was 34.4 ± 5.1 years, which was consistent with Ritch’s study [8]. Besides, they found PIS was the most common mechanism (35 patients, 52.2%) and many of them had an element of pupillary block. PIS refers to a postoperative condition in which a patent iridotomy has removed the relative pupillary block, but gonioscopically confirmed angle closure recurs without shallowing of the anterior chamber axially [11, 12]. The reason for angle closure due to PIS is the large and anteriorly inserted ciliary processes hold the iris root in opposition to the trabecular meshwork, easy to develop synechial angle closure, even after a successful iridotomy [13]. It was reported that PIS was seen more often in young adults compared with pupillary block, especially in 30–50 years old patients [11, 13, 14]. However, we found mixed mechanism (50 patients, 50.0%) was the most common type. This may be explained by the differences in ethnicity, since a previous study carried out in Chinese reported that more than half of the patients (54.8%) with PACG were caused by mixed mechanism [15]. Although the proportion of PIS (25%) in our study was not as high as previously reported in western populations, it was still higher than general PACG population in Asian [16]. In addition, compared with elder patients, our results showed a clear tendency toward shorter AL and shallower ACD of young PACG patients [17–21] (Table 3). Despite the thinner lens thickness [22], they still have relatively more crowded anterior chamber, which was possibly due to higher proportion of non-pupillary block mechanism in young PACG patients.

**Table 3:** Reported axial length and anterior chamber depth in our young PACG patients and in other PACG populations

	Number	Diagnosis	Age (years)	AL (mm)	ACD (mm)	LT (mm)
Our patients	100	PACG	25.6 ± 13.0	21.66 ± 1.03	1.89 ± 0.30	4.36 ± 0.36
Chen et al. [17]	90	PACG	66.0 ± 7.4	22.68 ± 0.80	2.32 ± 0.17	5.13 ± 0.36
Ngo et al. [18]	50	PACG	66.5 ± 9.2	22.89 ± 0.97	2.60 ± 0.25	4.66 ± 0.75
Nongpiur et al. [19]	111	PAC/PACG	65.4 ± 8.8	22.84 ± 0.96	2.66 ± 0.38	NA
Ozaki et al.[20]	109	PAC/PACG	73.5 ± 7.0	22.22 ± 0.77	2.51 ± 0.39	4.91 ± 0.54
Ho et al. [][21]	117	PAC/PACG	73.5 ± 7.1	22.20 ± 0.79	2.49 ± 0.05	4.94 ± 0.10

*PAC*, primary angle-closure; *PACG*, primary angle-closure glaucoma; *AL*, axial length; *ACD*, anterior chamber depth; *LT*, lens thickness

As we all know, family history is an important risk factor for PACG. Our study showed that 30.0% of young PACG patients had positive family histories and 70.8% of them were parents. The rate of family history was higher than general PACG populations (8.2%) [23] and general PAC populations (25.0%) [24], in which the mean diagnosed age was around 60 years. It seems that young PACG patients tend to have higher rate of glaucoma family history than general PACG patients.

Malignant glaucoma is one of the most challenging problems occurring after filtration surgery. Sixteen out of 100 patients (16.0%) in our study suffered from MG after trabeculectomy, which seems to be much more prevalent than the reported 2% incidence of MG after glaucoma surgery [25, 26]. A 5-year retrospective analysis in Chinese by Zhang et al. [26] showed MG accounted for 2.17% of 4640 PACG patients; the mean age of MG patients and total PACG patients were 49.67 ± 13.69 years and 62.34 ± 11.13 years respectively. Together with our results, it seems that there is a strong tendency of developing MG in young PACG patients. Moreover, compared with studies on general PACG patients [17–21] (Table 3), our patients had shorter ALs (21.66 ± 1.03 mm), and among them MG patients had even shorter ALs (21.29 ± 0.82 mm).

The second leading cause of ACG was uveitis (61 patients, 20.3%). This group of patients was male-dominated, which was similar to the report of Ritch et al. [8]. Previous studies demonstrated that open-angle glaucoma was the most common form of uveitic glaucoma [27, 28]. There are few studies reporting the incidence of ACG in patients with uveitis, except Takahashi et al. who evaluated 293 eyes with uveitic glaucoma, which found 7.5% of the eyes had PAS wider than 180°, 37.2% of the eyes had PAS less than 180°, and 55.3% of the eyes did not have PAS [29]. In our study, all the included patients had extensive PAS wider than 180° and most of them had complete angle closure. The substantial numbers of ACG patients secondary to uveitis suggested secondary ACG was an important complication of uveitis.

Patients with diagnosis of ASD comprises a variety of developmental conditions affecting the structures lying between the front surface of the cornea and the front surface of the vitreous, alone or in combination, or accompanied by systemic defects [30, 31]. Congenital anomalies typically include corneal opacity, posterior embryotoxon, iris hypoplasia, corectopia or polycoria, and adhesions between the iris and cornea or lens and cornea [31, 32]. Six types of ASD were seen in our patients, which included A-R syndrome, Peter’s anomaly, microcornea, aniridia, congenital ectropion uvea, and unclassified ASD. Unclassified ASD referred to patients having signs of ASD but could not fit into any specific category and most of them were babies with limited clinical information. The most common type was A-R syndrome in our study, which was consistent with a previous study stating that A-R syndrome was the most common type in ASD [31]. Previous studies showed that ASD conferred a 50% or greater risk of developing glaucoma, usually in adolescence or early adulthood [33]. Consistently, 33 out of 45 ASD patients were younger than 20 years old in our study. Glaucoma secondary to ASD can be caused by the following mechanisms: (1) open-angle glaucoma due to maldevelopment of the trabecular meshwork and Schlemm’s canal, and (2) ACG due to high insertion of the iris root over the trabecular meshwork or secondary PAS [34–37]. Apparently, since patients with extensive PAS and closed anterior chamber angle were included, our patients all belong to the second category of mechanisms.

The above three causes were the main components of the etiologies, which accounted for more than two-thirds (67.8%) of total patients. Diagnosed age distribution analysis showed that almost all PACG patients were older than 20 years old and most of them were between 31 and 40 years old. The prevalence of PACG among those patients aged over 30 years old was fourfold higher than patients under 30 years old, while patients younger than 20 years old were more likely to be ASD, especially patients with age younger than 10 years old. Thus, although there could be some PACG patients younger than 40 years old, diagnoses should be very careful if patients were younger than 30 years old; instead, ASD should be considered as the primary possible diagnosis.

Besides, nanophthalmos is another important cause of ACG in young patients. Nanophthalmos is characterized by a short AL, shallow anterior chamber, high lens/eye volume ratio, short corneal diameter, thickened sclera, and high hyperopia [38]. The mean age of nanophthalmos was 29.5 ± 8.8 years (range,13–40 years) and the mean AL was 18.03 ± 1.63 mm (range,14.57–19.92 mm) in our study. Similarly, the mean age was 28.7 ± 4.9 years (range, 23–32 years) and the AL ranged from 17.0 to 20.25 mm in Ritch’s study [8]. Another study reported by Kocak et al. [10] showed that the mean age of nanophthalmos patients with ACG was 14.25 years (7–29 years), and the average AL was 16.10 mm (range,14.30–19.33 mm). Our results and these studies suggested the shorter the AL, the younger the age of diagnosis. The majority of cases were sporadic in our study, although an autosomal recessive pattern of inheritance and occasional autosomal dominant cases have been reported [39, 40].

Furthermore, a growing body of research suggested bestrophinopathy [41] and other retinal diseases such as RP [42], congenital retinoschisis [43], FEVR [44], congenital retinal folds [45], Coat’s disease [46] were related to ACG. We have nine etiologies about retinal diseases related to ACG, they were RP, bestrophinopathy, PFV, congenital retinoschisis, ROP, FEVR, congenital retinal folds, Coat’s disease, and NVG due to RVO, PDR, and RD, altogether 35 patients (11.7%). Other reported etiologies such as Turner syndrome [47], Noonan syndrome [48], Alagille syndrome [49], childhood cystinosis [50], and drug-related [51] were not found in our patients.

The limitation of this study is its retrospective nature. The analysis of etiologies and clinical characteristics was based on medical charts’ review. Some of the records had insufficient information or incomplete examinations, which could cause bias in statistical analysis.

In summary, we have described the uncommon presentation of ACG in Chinese young patients, on whom the etiologies and the clinical characteristics were analyzed carefully. PACG, uveitis, and ASD were the most common causes. Patients diagnosed as PACG were mainly older than 30 years of age. They had higher ratio of glaucoma family history, shorter AL, shallower ACD, and higher risk of developing MG after trabeculectomy compared with elder PACG patients. ASD was the top reason of ACG in patients younger than 20 years old. It is noteworthy that retinal diseases such as RP, bestrophinopathy, PFV, congenital retinoschisis, ROP, FEVR, congenital retinal folds, and Coat’s disease could also be related to ACG. In brief, angle closure in young patients is unusual. Diagnosis should be made very prudently on this group of patients. Since most of the etiologies could be identified precisely, more careful and comprehensive examination is needed for early detection and proper treatment for young ACG patients.

## Supplementary Information

Below is the link to the electronic supplementary material.

Supplementary file1 (XLSX 92.6 KB)
