# Correction to: Etiologies and clinical characteristics of young patients with angle-closure glaucoma: a 15-year single-center retrospective study

**DOI:** 10.1007/s00417-021-05260-7

**Published:** 2021-07-02

**Authors:** Feng Gao, Jiajian Wang, Junyi Chen, Xiaolei Wang, Yuhong Chen, Xinghuai Sun

**Affiliations:** 1 Department of Ophthalmology & Visual Science, Eye & ENT Hospital, Shanghai Medical College, Fudan University, 83 Fenyang Road, Shanghai, 200031 China; 2 NHC Key Laboratory of Myopia, Chinese Academy of Medical Sciences, and Shanghai Key Laboratory of Visual Impairment and Restoration (Fudan University), Shanghai, China; 3 State Key Laboratory of Medical Neurobiology and MOE Frontiers Center for Brain Science, Institutes of Brain Science, Fudan University, Shanghai, China

**Correction to: Graefe’s Archive for Clinical and Experimental Ophthalmology**

10.1007/s00417-021-05172-6

The original version of this article inadvertently contained mistakes in Table 1. This is being corrected in this publication.

**Table 1:** The demographic information of our patients

Diagnosis	Number ofPatients	Age, Mean ± SD(range), years	GenderFemale/Male	Affected eyesUnilateral /Bilateral
PACG	100	34.4 ± 5.1 (13-40)	68/32	16/166
Uveitis	61	23.8 ± 12.8 (0.25-40)	20/41	47/28
ASD	45	11.5 ± 12.6 (0.13-38)	14/31	21/48
ICE syndrome	22	33.2 ± 6.3 (14-40)	14/8	20/4
NVG	15	20.1 ±12.2(0.1-40)	5/10	15/0
Nanophthalmos	13	29.5 ± 8.8 (13-40)	6/7	6/14
RP	9	29.7 ± 7.0 (18-39)	4/5	0/18
Spherophakia	8	13.4 ± 5.6 (6-20)	4/4	1/14
Bestrophinopathy	6	26.2 ± 6.4 (18-34)	5/1	0/12
PFV	6	19.9 ± 16.4 (0.3-39)	3/3	4/4
Iridociliary cysts	4	24 ± 14.0 (8-40)	3/1	3/2
Congenital retinoschisis	2	19 ± 1.4 (18-20)	0/2	0/4
Marfan’s syndrome	1	40	1/0	0/2
ROP	1	17	1/0	1/0
FEVR	1	12	0/1	1/0
Congenital retinal folds	1	30	1/0	1/0
Coat’s disease	1	3	0/1	1/0
Neurofibromatosis	1	4	0/1	1/0

*PACG*, Primary angle-closure glaucoma; *ASD*, anterior segment dysgenesis; *ICE*, iridocorneal endothelial; *NVG*, neovascular glaucoma; *RP*, retinitis pigmentosa; *PFV*, persistent fetal vasculature;* ROP*, retinopathy of prematurity; *FEVR*, familial exudative vitreoretinopathy. Seven patients (6 females and 1 male) were unknown due to incomplete information.

The original article has been corrected.

